# Preparation of Ti_3_C_2_T_x_/NiZn Ferrite Hybrids with Improved Electromagnetic Properties

**DOI:** 10.3390/ma13040820

**Published:** 2020-02-11

**Authors:** Xiaobing Zhou, Youbing Li, Qing Huang

**Affiliations:** Engineering Laboratory of Advanced Energy Materials, Ningbo Institute of Materials Technology and Engineering, Chinese Academy of Sciences, Ningbo 315201, China; liyoubing@nimte.ac.cn (Y.L.); huangqing@nimte.ac.cn (Q.H.)

**Keywords:** MXenes, ferrite, electric current field-assisted sintering technology, electromagnetic property

## Abstract

Ti_3_C_2_T_x_/NiZn ferrite composites were synthesized using a co-precipitation hydrothermal method, and further consolidated using electric current field-assisted sintering technology. Nano NiZn ferrites were inserted into the Ti_3_C_2_T_x_ interlayers with uniform coverage on their surfaces. The incorporation of MXenes promoted the sintering kinetics of the NiZn ferrite ceramics. The electrical conductivity increased by six orders of magnitude compared to pure NiZn ferrite ceramics at room temperature. The present work provides a potential way to develop a large family of dense MXenes/ferrite multiphase ceramics. The multiphase ceramics could be potentially used for the on-beam-line higher-order mode load in advanced particle accelerators.

## 1. Introduction

Spinel NiZn ferrites have been extensively studied because of their suitable magnetic properties, high Curie temperature, and good electromagnetic wave absorbing performance [1,2,3,4]. One of the most important applications of ferrites is as an on-beam-line higher-order mode (HOM) load in advanced particle accelerators [5,6]. The electromagnetic wave absorption materials employed in this area need to satisfy some harsh requirements, such as low vacuum out-gassing rate (high density), appropriate direct-current (DC) electrical conductivity for charge drainage at low temperatures, good thermal conductivity, and wide-range-bandwidth microwave absorption [7]. On the other hand, NiZn ferrites exhibit insulating behavior at low temperatures, making them unsuitable for charge drainage.

Naguib et al. [8] discovered MXenes, a new family of two-dimensional layered structural transition metal carbides and/or nitrides. MXenes are prepared by etching away the A layer atoms from the corresponding ternary ceramics of MAX phases by hydrofluoric acid (HF), where “M” represents a transition metal, “A” is usually a III A or IVA element (such as Al, Si, Ge, or Ga), and “X” is C and/or N. Generally, MXene surfaces are terminated with abundant -OH, =O, and/or -F surface groups after HF etching [8]. The formula, M_n+1_X_n_T_x_, was suggested to be more accurate, where n = 1 to 3, and T is the surface terminated groups. The MXene family has attracted increasing attention because of their excellent metallic conductivity, high specific surface area, and hydrophilicity. MXenes are considered promising candidates for electrodes in supercapacitors, anode materials for Li-batteries, heavy metal ions adsorbents, etc. [9,10,11,12,13,14,15,16,17]. In addition, the excellent microwave absorbing performance of Ti_3_C_2_T_x_ MXenes was revealed. Han et al. [18] examined the electromagnetic loss mechanisms of Ti_3_C_2_T_x_ before and after annealing. The formation of a localized sandwich structure containing TiO_2_ nanocrystals and amorphous carbon without sacrificing the original 2D layer structure was considered the main reason. Shahzad et al. [19] first fabricated highly flexible MXene films and nacre-like MXene-sodium alginate (SA) composite films for EMI shielding. The highest EMI shielding effectiveness of 92 dB (45 μm thick Ti_3_C_2_T_x_ film) among the synthetic materials of comparable thickness produced to date was developed. They attributed the high EMI shielding performance to the excellent electrical conductivity of the Ti_3_C_2_T_x_ films (4600 S/cm) and the multiple internal reflections from Ti_3_C_2_T_x_ flakes in free-standing films. In our previous work, a Ti_3_C_2_T_x_/ferrite composite with a 5 wt.% Ti_3_C_2_T_x_ MXenes loading exhibited high reflection loss (−42.5 dB) at 13.5 GHz [20]. 

Therefore, MXenes have potential applications in the microwave absorption area. The excellent metallic conductivity, 2D layer structure, and good hydrophilicity with abundant negative termination groups make Ti_3_C_2_T_x_ a potential filler of ferrite to improve the electrical conductivity. Furthermore, to obtain a highly dense Ti_3_C_2_T_x_/ferrite composite, an electric current field-assisted sintering technology (FAST) was used [21,22,23]. The high-density electric current can promote mass diffusion and enable the fabrication of dense materials in a short time at low sintering temperatures compared to conventional methods [24]. In this study, a co-precipitation hydrothermal method was used to synthesize a novel Ti_3_C_2_T_x_/NiZn ferrite composite. Furthermore, the as-received Ti_3_C_2_T_x_/NiZn ferrite powders were sintered by FAST. The phase, microstructure, morphology, and electromagnetic properties were examined. The electrical conductivity of the fabricated ceramics was measured from 100 to 300 K. To the best of the authors’ knowledge, this is the first effort to fabricate dense Ti_3_C_2_T_x_/NiZn ferrite multiphase ceramics by FAST. The present communication provides a way to develop a large family of dense MXenes/ferrites multiphase ceramics for electromagnetic devices applications.

## 2. Experimental Procedure

### 2.1. Materials and Experiments

The Ti_3_C_2_T_x_ powders were prepared using the procedure reported by Naguib et al. [8]. A 1 g sample of Ti_3_AlC_2_ powder was etched by stirring lightly every 12 h in a 10 mL HF solution (40 wt.%) for 72 h at room temperature. The resulting slurry was dried at 30 °C for 48 h after centrifuging and washing several times with deionized water and alcohol. To improve the dispersion in water, the as-prepared Ti_3_C_2_T_x_ powders were functionalized by adding 5 wt.% sodium lignin sulfonate (SLS) and followed by ultrasonic dispersing process for 45 min. A 1 mg/mL SLS-functionalized Ti_3_C_2_T_x_ dispersion solution was obtained after removing the excessive SLS by filtration and washing. Subsequently, the appropriate SLS-functionalized Ti_3_C_2_T_x_ suspension was mixed with a solution containing Ni(NO_3_)_2_, Zn(NO_3_)_2_, and Fe(NO_3_)_3_ with a Ni:Zn:Fe molar ratio of 0.5:0.5:2 (Table 1). The above solution mixture was added dropwise to NaOH solution with vigorous stirring until the pH reached 10.5 ± 0.1. The as-prepared precursor was then transferred to a Teflon-lined autoclave and treated hydrothermally at 200 °C for 2 h. The resulting precipitates were then washed several times with deionized water and ethanol. The Ti_3_C_2_T_x_/NiZn ferrite powders were obtained after drying at 80 °C for 5 h. The Ti_3_C_2_T_x_/NiZn ferrite composites were sintered further by FAST (HP D25, FCT Systeme GmbH, Effelder-Rauenstein, Germany) in an argon gas atmosphere. Ti_3_C_2_T_x_/NiZn ferrite composite ceramics were fabricated at a sintering temperature of 800 °C for 5 min with heating and cooling rates of 100 °C/min. A 35 MPa axial pressure was applied during sintering. For comparison, pure NiZn ferrite without Ti_3_C_2_T_x_ was prepared using the same method.

### 2.2. Characteristics

The phases of all specimens were detected by X-ray diffraction (XRD, D8 Advance, Bruker AXS, Bremen, Germany) using Cu Kα radiation at a step size of 0.02°/2θ with a collection time of 1 s per step. An operating voltage of 40 kV and a current 35 mA were used. The scanning was done in a 2θ range of 5°–70°. The microstructure was observed by scanning electron microscopy (SEM, Quanta 250 FEG, FEI, Hillsboro, OR, USA) equipped with an energy dispersive spectroscopy (EDS) system. The resistivities of samples were measured using a physical properties measurement system (PPMS, Quantum Design, San Diego, CA, USA) at room temperature. The electrical conductivities were then calculated. The magnetic hysteresis loops were collected using a vibrating sample magnetometer (PPMS-VSM, Quantum Design, San Diego, CA, USA) with a maximum magnetic field of 30 kOe at room temperature.

## 3. Results and Discussion

### 3.1. Phase and Microstructure Morphology of Ti_3_C_2_T_x_/NiZn Ferrite Powders

**Figure 1:**
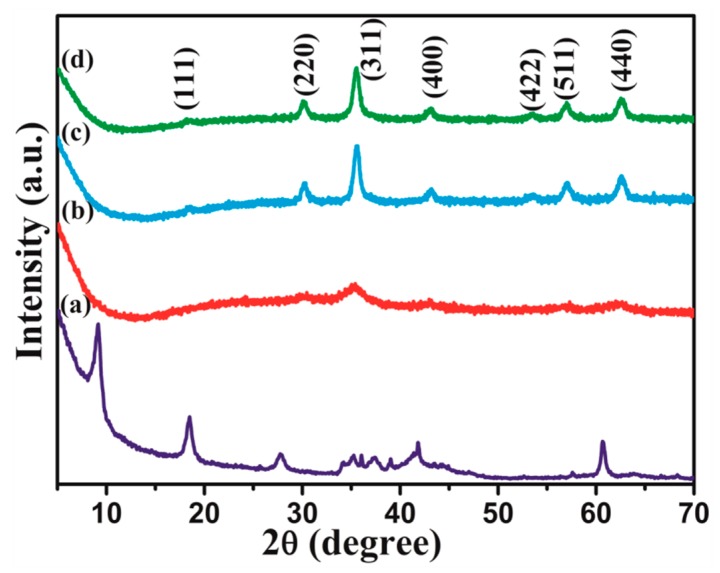
XRD patterns of (**a**) Ti_3_C_2_T_x_ MXenes, (**b**) precursor of 2 wt.% Ti_3_C_2_T_x_/NiZn ferrite, precursors hydrothermally treated at 200 °C for 2 h: (**c**) Ni_0.5_Zn_0.5_Fe_2_O_4_, (**d**) 2 wt.% Ti_3_C_2_T_x_/NiZn ferrite.

Figure 1a presents the XRD patterns of the Ti_3_C_2_T_x_ MXenes. The Ti_3_C_2_T_x_ MXenes and Ti_3_AlC_2_ were detected. The relative diffraction peak intensity of Ti_3_AlC_2_ (104) at 39° 2θ was obviously decreased, indicating that Ti_3_C_2_T_x_ MXenes had been formed with the Al atomic layers exfoliated. The typical peaks of Ti_3_C_2_F_2_ were detected at 8.9° and 18.4° 2θ, showing that F was terminated on the Ti_3_C_2_T_x_ surface. The peak at 27.4° 2θ was assigned to Ti_3_C_2_(OH)_2_ [8]. Therefore, Ti_3_C_2_T_x_ MXenes surfaces were terminated -OH, and –F groups after HF etching. These abundant negative groups combined with the typical 2D layer structure of Ti_3_C_2_T_x_ MXenes provide natural channels for the in-situ intercalation of Ni, Zn, and Fe ions. Figure 1b–d shows XRD patterns of the precursor of 2 wt.% Ti_3_C_2_T_x_/NiZn ferrite and after a hydrothermal treatment at 200 °C for 2 h. The precursor exhibited poor crystallinity with a broad peak (311) (Figure 1b), while the precursor transformed completely to the Ni_0.5_Zn_0.5_Fe_2_O_4_ spinel phase after the hydrothermal reaction at 200 °C for 2 h (Figure 1c,d). All typical peaks of 2 wt.% Ti_3_C_2_T_x_/NiZn ferrite (Figure 1d) corresponded well to the Joint Committee on Powder Diffraction Standards (JCPDS) card NO. 08-0234, indicating that it is a pure spinel phase. The incorporation of Ti_3_C_2_T_x_ did not affect the formation of the designed NiZn ferrite phase. Naguib et al. [25] and Gao et al. [26] treated Ti_3_C_2_T_x_ hydrothermally in water at 180–200 °C, and detected a TiO_2_ (anatase) phase. In the present work, neither Ti_3_C_2_T_x_ nor TiO_2_ characteristic peaks were detected, which may be due to the low content of Ti_3_C_2_T_x_ fully coated with NiZn ferrite nanoparticles.

**Figure 2:**
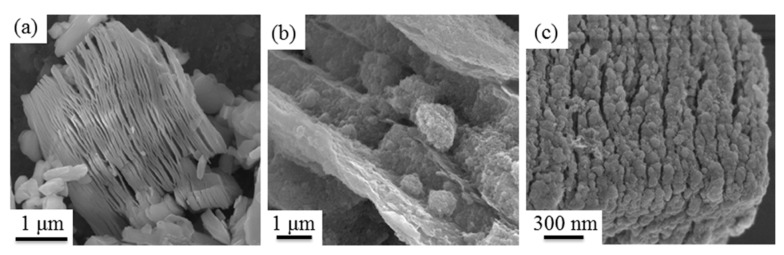
SEM images of (**a**) pure Ti_3_C_2_T_x_ MXenes, (**b**) precursor of 2 wt.% Ti_3_C_2_T_x_/NiZn ferrite composites, and (**c**) after hydrothermal treated at 200 °C of precursor of 2 wt.% Ti_3_C_2_T_x_/NiZn ferrite composites.

SEM was carried out to verify the NiZn ferrite nanoparticles inserted into the interlayer of Ti_3_C_2_T_x_ MXenes. Figure 2a presents a typical SEM image of pure Ti_3_C_2_T_x_ MXenes. The 2D-layered stacked sheets appeared like an accordion due to the Al atomic layers of Ti_3_AlC_2_ eliminated after HF etching. Figure 2b presents an SEM image of the 2 wt.% Ti_3_C_2_T_x_/NiZn ferrite composites precursor. The nanoparticles were inserted into the interlayer and coated on the surface of Ti_3_C_2_T_x_ MXenes. As described above, the modified Ti_3_C_2_T_x_ MXenes have abundant negative surface charges, which can electrostatically attract positively charged Ni^2+^, Zn^2+^, and Fe^3+^ hydroxide during the co-precipitation process. Therefore, after the hydrothermal reaction at 200 °C, the nanoparticles crystallized gradually with in-situ grain growth on the surface of Ti_3_C_2_T_x_ MXenes (Figure 2c). The EDS analysis (not shown here) of the nanoparticles in Figure 2b,c revealed mainly O, Ni, Zn, and Fe. Combined with the XRD analysis results, it is not difficult to confirm that the nanoparticles were NiZn ferrites. NiZn ferrite nanoparticles agglomerated due to the magnetic dipole interactions and high surface energy. Furthermore, Ti_3_C_2_T_x_ MXenes still maintained the layered structure.

### 3.2. Phase and Microstructure Morphology of Ti_3_C_2_T_x_/NiZn Ferrite Ceramics

**Figure 3:**
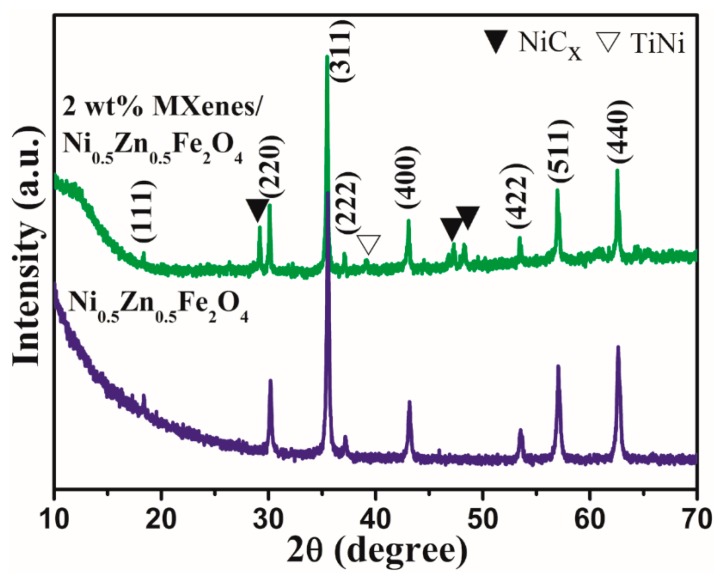
XRD patterns of pure NiZn ferrite and 2 wt.% Ti_3_C_2_T_x_/NiZn ferrite composites after sintering at 800 °C by FAST.

Figure 3 presents XRD patterns of pure NiZn ferrite and 2 wt.% Ti_3_C_2_T_x_/NiZn ferrite composites after sintering at 800 °C by FAST. Compared to the Ti_3_C_2_T_x_ free sample, NiC_x_ (JCPDS NO. 45-0979) and TiNi alloy (JCPDS NO. 27-0344) impure phases were detected in the 2 wt.% Ti_3_C_2_T_x_/NiZn ferrite ceramics. This may be due to the carbo-thermal reduction reaction at the Ti_3_C_2_T_x_ and NiZn ferrite interface. A similar interfacial reaction was observed in the CNT/ferrite composites [7]. On the other hand, as reported by Li et al. [27], Ti_3_C_2_T_x_ MXenes were oxidized completely and the anatase phase transformed fully to rutile in the oxygen atmosphere at 1000 °C while neither TiC nor TiO_2_ was detected in the present work. This may be due to the low content of Ti_3_C_2_T_x_ introduced. The crystallite sizes of the Ni_0.5_Zn_0.5_Fe_2_O_4_ and Ti_3_C_2_T_x_/Ni_0.5_Zn_0.5_Fe_2_O_4_ composites were determined from the XRD patterns using Scherrer’s equation [28]:

D = kλ/βcosθ(1)

where D is the mean crystallite size, k is a constant (0.89), λ is the wavelength of X-rays and equal to 0.154056 nm, β is the full width at half maxima (FWHM) measured in radians and θ is the diffraction angle. The mean crystallite sizes of pure Ni_0.5_Zn_0.5_Fe_2_O_4_ and 2 wt.% Ti_3_C_2_T_x_/ Ni_0.5_Zn_0.5_Fe_2_O_4_ composites were 89.3 and 179.5 nm, respectively.

**Figure 4:**
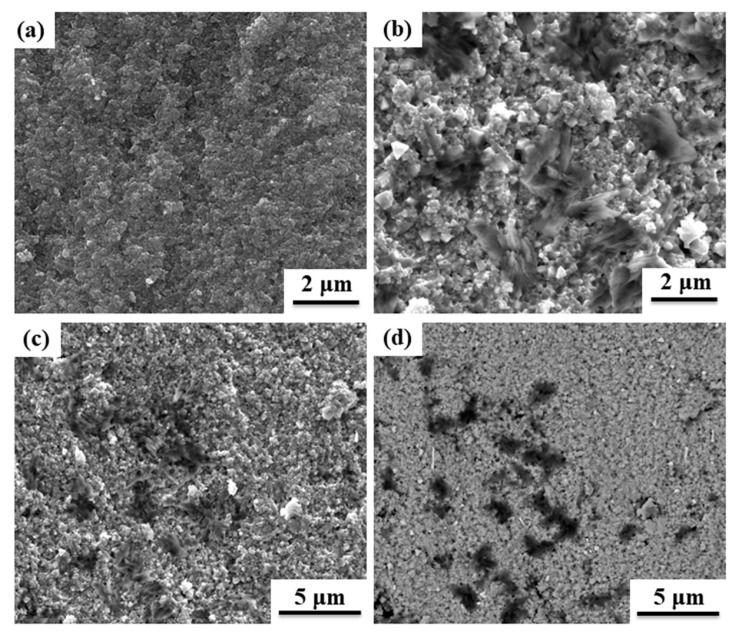
SEM images of the fracture surface of (**a**) pure NiZn ferrite, (**b**) 2 wt.% Ti_3_C_2_T_x_/NiZn ferrite composites, low magnification SEM images of 2 wt.% Ti_3_C_2_T_x_/NiZn ferrite composites (**c**) secondary electron image, and (**d**) backscattered electron image (the same area of **c**).

Figure 4a,b presents SEM images of the fracture surfaces of pure NiZn ferrite and 2 wt.% Ti_3_C_2_T_x_/NiZn ferrite composites, respectively. Some pores were observed in the pure NiZn ferrite because of the poor consolidation. On the other hand, it was difficult to find obvious pores in the 2 wt.% Ti_3_C_2_T_x_/NiZn ferrite composites. To further observe the Ti_3_C_2_T_x_ distribution in the 2 wt.% Ti_3_C_2_T_x_/NiZn ferrite composites, low magnification SEM images (both the backscattered and secondary electrons image) are shown in Figure 4c,d. The dark area corresponds to Ti_3_C_2_ due to the low Z contrast. EDS analysis indicated that the layered structure grain was composed of Ti, C, and O (data not shown). The Ti_3_C_2_T_x_ distributed in the NiZn ferrite matrix, while some area was rich Ti_3_C_2_T_x_. Ti_3_C_2_T_x_ still retained its layered structure after sintering at 800 °C by FAST (Figure 4b,c). On the other hand, some dispersed nanosize “white” spherical grains (high Z contrast phase) were observed, which may be the TiNi alloy according to EDS (data not shown).

The apparent density of the bulk ceramics was measured using the Archimedes technique. The relative densities of pure NiZn ferrite and composites were 92.4% and 98.9%, respectively. The theoretical densities of NiZn ferrite and Ti_3_C_2_T_x_ were approximately 5.30 [29] and 4.22 g/cm^3^ (calculated using the approximate lattice parameters of Ti_3_C_2_O_2_; a = 3.04 Å and c = 9.83 Å), respectively. This indicates that the introduction of the Ti_3_C_2_T_x_ can promote the sintering of ferrite ceramics. The heating principle of FAST is the Joule heat generated by the high-density currents flowing through the powders and/or the graphite die. Because pure NiZn ferrite powders are poor electrical conductors, they were heated mostly by absorbing the heat generated from the graphite die. The conductive network could be formed by the introduction of Ti_3_C_2_T_x_ because the electrical conductivity of Ti_3_C_2_T_x_ (4600 S/cm) [19] is much higher than that of pure ferrite (~10^−6^ S/cm) [7]. Therefore, a part of the high-density pulsed current could have flowed directly through the composites. The local temperature in the interface of Ti_3_C_2_T_x_ and NiZn ferrite should be higher than that detected by the upside optical thermometer. The as-formed Ti_3_C_2_T_x_ conductive network acted as a “susceptor” and promoted sintering. On the other hand, the local temperature gradient led to carbo-thermal reduction at the Ti_3_C_2_T_x_ and NiZn ferrite interface.

### 3.3. Electromagnetic Properties

**Figure 5:**
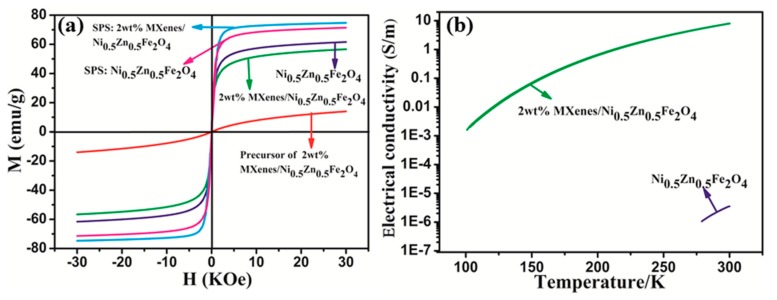
(**a**) Hysteresis loops of precursor, powders, and bulks of pure ferrite and 2 wt.% Ti_3_C_2_T_x_/NiZn ferrite composites, (**b**) temperature dependence of the electrical conductivity of pure ferrite and 2 wt.% Ti_3_C_2_T_x_/NiZn ferrite composites.

The magnetic performance of the precursor, powders, and bulk of pure ferrite and 2 wt.% Ti_3_C_2_T_x_/NiZn ferrite composites were investigated at room temperature. Figure 5a presents the corresponding hysteresis loops. The residual magnetization and coercive force of all samples were almost zero, suggesting that they correspond to the soft ferrite character. The saturation magnetization (M_s_) of the precursor of 2 wt.% Ti_3_C_2_T_x_/NiZn ferrite composites was only 14.1 emu/g due to the poor crystallinity. After the hydrothermal treatment at 200 °C for 2 h, the M_s_ of 2 wt.% Ti_3_C_2_T_x_/NiZn ferrite powders increased to 56.6 emu/g. This was attributed to the amorphous precursor transferred to the pure spinel phase. Compared to the pure NiZn ferrite powders (61.6 emu/g), this is slightly lower due to the incorporated nonmagnetic Ti_3_C_2_T_x_. The M_s_ of the bulk NiZn ferrite and the 2 wt.% Ti_3_C_2_T_x_/NiZn ferrite composites were increased further to 71.4 and 74.7 emu/g, respectively, after sintering by FAST at 800 °C. The M_s_ of the 2 wt.% Ti_3_C_2_T_x_/NiZn ferrite composites was slightly higher than that of NiZn ferrite ceramics. The effects of the M_s_ of 2 wt.% Ti_3_C_2_T_x_/NiZn ferrite composites are relatively complex. According to phase analysis, a portion of Ni precipitated from the 2 wt.% Ti_3_C_2_T_x_/NiZn ferrite composites to form NiC_x_ and TiNi alloy impurity phases, which would decrease the M_s_. In addition, NiZn ferrite is a mixed spinel structure with a chemical formula of (Zn_1−x_Fe_1−y_)[Ni_x_Fe_1+y_]O_4_, in which Zn^2+^ ions prefer to occupy the A-sites (tetrahedral sites) due to the stable sp3 hybrid configuration, while Ni^2+^ ions primarily to occupy the B-sites (octahedral sites) owing to the best fit charge distribution in the octahedral crystal field [30,31]. The ZnFe_2_O_4_ (x = 0, y = 1) is a normal spinel ferrite [4], while NiFe_2_O_4_ (x = 1, y = 0) is an inverse spinel ferrite. Jadhav et al. [32] reported that the M_s_ of Ni_x_Zn_1-x_Fe_2_O_4_ (x = 0.2–0.8) was significantly influenced by the distribution of cations at the lattice sites and the resultant inter-sub-lattice and intra-sub-lattice exchange coupling interactions. The M_s_ increased with the increase in Ni concentration until the maximum magnetization was detected for the sample of Ni_0.5_Zn_0.5_Fe_2_O_4_ [32]. Kumar et al. observed similar phenomena in Ni_x_Zn_1−x_Fe_2_O_4_ (0.1 ≤ x ≤ 0.5) too [33]. Therefore, the Ni precipitation from the 2 wt.% Ti_3_C_2_T_x_/NiZn ferrite composites would decrease the Ni concentration in NiZn ferrite, resulting in the decrement of M_s_. Furthermore, the demagnetization field generated by the incorporation of nonmagnetic Ti_3_C_2_T_x_ would also decrease M_s_. On the other hand, the high density and large crystallite size have positive effects on improving M_s_. The relative density of the 2 wt.% Ti_3_C_2_T_x_/NiZn ferrite composites (98.9%) is much higher than that of pure NiZn ferrite (92.4%). Furthermore, the mean crystallite size of 2 wt.% Ti_3_C_2_T_x_/NiZn ferrite composites was approximately 179.5 nm, which is almost twice higher than that of the pure NiZn ferrite (89.3 nm). Therefore, the high density and large crystallite size seem to be dominant factors for the magnetic properties in the present work.

Figure 4b shows the temperature dependence of the electrical conductivity of the pure ferrite and 2 wt.% Ti_3_C_2_T_x_/NiZn ferrite composites after sintering at 800 °C by FAST. The pure NiZn ferrite showed typical insulating characteristics because the electrical conductivity was only 3.5 × 10^−6^ S/m at room temperature and 1.1 × 10^−6^ S/m at 278 K. With the incorporation of 2 wt.% Ti_3_C_2_T_x_, the electrical conductivity increased significantly. The electrical conductivity reached 8.1 S/m at room temperature, which is a six order of magnitude improvement compared to that of pure NiZn ferrite ceramics. The electrical conductivity decreased gradually with decreasing temperature, exhibiting semi-conductor behavior. When the temperature was decreased to 100 K, the electrical conductivity still remained at 1.6 × 10^−3^ S/m. The sensitive influence of Ti_3_C_2_T_x_ incorporation on the electrical conductivity may be due to two reasons. First, it is due mainly to the excellent electrical conductivity of Ti_3_C_2_T_x_, which had been predicted theoretically and proven experimentally. Lukatskaya et al. [10] performed a simulation study using density functional theory, and suggested that Ti_3_C_2_T_x_ would show metallic-like electrical conductivity behavior because of the high electron density of states near the Fermi level [N(E_f_)]. Shahzad et al. [19] examined the electrical conductivity of Ti_3_C_2_T_x_ films, which was as high as 4600 S cm^-1^. Second, the hydrothermal in-situ co-precipitation method used in the present study is beneficial for Ti_3_C_2_T_x_ flakes to disperse uniformly in the ferrite matrix. The uniform dispersion of Ti_3_C_2_T_x_ flakes formed three-dimensional (3D) conductive networks and provided charge carriers with a more conductive path. Furthermore, FAST decreased the sintering temperature, which was good for protecting Ti_3_C_2_T_x_ 3D conductive networks. Ti_3_C_2_T_x_ was still subject to possible oxidation to form TiO_2_ and amorphous graphite, which has a positive effect on improving the electrical conductivity [18]. The high electrical conductivity at a low temperature is beneficial to charge drainage when used as a HOM load in advanced accelerators.

## 4. Conclusions

Highly dense (98.9% relative density) Ti_3_C_2_T_x_/NiZn ferrite multiphase ceramics were first developed by FAST. Ti_3_C_2_T_x_ MXenes with high electrical conductivity act as susceptors and promote the consolidation of the composites. The saturation magnetization of 2 wt.% Ti_3_C_2_T_x_/NiZn ferrite (74.7 emu/g) was higher than that of the pure ferrite ceramics (71.4 emu/g) due to the high relative density and large grain size. Furthermore, the electrical conductivity increased six orders of magnitude compared to the pure NiZn ferrite ceramics at room temperature. The present work may open the door to the development of a large family of highly dense MXenes/ferrites multiphase ceramics for electromagnetic devices applications.

## Figures and Tables

**Table 1:** Characteristics of the synthesized samples.

Sample	Molar Ratio Ni(NO_3_)_2_: Zn(NO_3_)_2_: Fe(NO_3_)_3_	Molar Ratio NO_3_^−^: OH^−^	Weight Present of Ti_3_C_2_T_x_ (wt.%)
Ni_0.5_Zn_0.5_Fe_2_O_4_	0.5:0.5:2	1:1	0
2 wt.% Ti_3_C_2_T_x_/ Ni_0.5_Zn_0.5_Fe_2_O_4_	0.5:0.5:2	1:1	2
